# The morning after the night before: Alcohol-induced blackouts impair next day recall in sober young adults

**DOI:** 10.1371/journal.pone.0250827

**Published:** 2021-05-03

**Authors:** Judith Jackson, David I. Donaldson, Benjamin Dering

**Affiliations:** Psychology Division, Faculty of Natural Sciences, University of Stirling, Stirling, Scotland, United Kingdom; University of Sydney, AUSTRALIA

## Abstract

Binge-drinking in adolescents and young adults is a widespread problem, however, an often unreported consequence of binge-drinking behaviour is an alcohol-induced memory blackout (MBO). An MBO is a transient amnesic event resulting from rapid, excessive alcohol consumption. Here, we examine the short-term impact of an alcohol-induced MBO event (testing < 20 hours after blackout) on memory performance in people who have experienced a high volume of MBOs. In addition, we aimed to test the hypothesis that people who experience a high volume of MBOs may have poorer recall than non-blackout controls in either sober or intoxicated states. Three episodic memory paradigms consisting of free recall, serial recall, and depth of encoding tasks, were conducted by a group of alcohol drinkers who had never experienced a memory blackout, and those who reported at least 9 in the preceding 12-months. Studies were completed sober and after alcohol by all participants, and sober but after blackout by the experimental group. Accuracy of recall was assessed with linear mixed effects modelling for all experiments and conditions. Recall rate both before and after alcohol consumption was similar between groups, with poorer recall after drinking alcohol by all participants in all three studies. After blackout, MBO participants showed no significant improvement from their intoxicated state in serial recall and depth of encoding tasks, but an improvement in free recall. Further analysis of these findings revealed that 10 out of 23 participants showed significantly impaired performance after blackout during free recall, extending up to 17 participants in serial recall. In general, alcohol reduced recall rate in both blackout and control participants similarly, but recall following MBO remained poor. Our evidence suggests that alcohol-induced blackouts impair memory functioning the next day, and future research should establish the duration of deficits after an acute alcohol-induced blackout episode.

## Introduction

An alcohol-induced memory blackout (MBO) is a transient amnesic event during which the individual remains conscious in the environment but loses the capacity to form long term episodic memories (i.e., memories for lived events and experiences). They are elicited by binge-drinking causing a rapid spike in blood alcohol content. Binge-drinking within adolescence and young adults is accepted as a global problem [1–4], yet the immediate consequences of binge-drinking, which can lead to an MBO, are rarely discussed. In sum, the long-term damage to people engaging in binge-drinking practices may in part be attributable to the frequency of MBOs experienced, i.e., that blackout events can be considered a marker of extreme alcohol binge-drinking, which in turn could inhibit memory and cognitive functioning more than average levels of alcohol consumption. Thus, the aims of the present paper are to [1] identify whether young adults who experience a high volume of MBOs are poorer in terms of episodic memory performance compared to non-blackout controls, either when sober or after ingesting alcohol, and [2] assess whether memory performance remains impaired the day after an alcohol-induced blackout, in sober young adults.

An MBO occurs when a rapid rise in blood alcohol levels disrupts processing within the hippocampus [5]. As well as reducing cortical activity (through the known actions of alcohol on Glutamatergic and GABAergic neurons), alcohol leads to the inhibition of CA1 pyramidal neurons [6], likely disrupting the transfer of information from short to long term storage, and as a result, the ability to retain new memories is restricted. Two types of MBO have been identified–*fragmentary* and *enbloc* [7]. The term fragmentary blackout describes the more commonly experienced type of MBO, where episodic memory is punctuated by brief periods of memory loss. Some recovery of episodes has been observed in people after experiencing a fragmentary blackout, yet this often follows from cues by peers [7]. In contrast, an enbloc blackout could be described as a complete inability to form any new memories over an extended period of time, with no recovery of any episodes. There is a dose dependent relationship between alcohol and MBOs, with fragmentary blackouts not normally reported in levels of less than 0.06% BAC, while enbloc blackouts are typically reported following higher blood alcohol levels than a fragmentary blackout [8].

Interestingly, not all heavy drinkers experience blackouts [7, 9], and it is known that a wide range of factors influence when, or even if they occur. For example, the quantity of alcohol consumed and speed of drinking [5, 10], gender [11], physiological differences [7, 9], environmental influences [12] and genetics [9, 11], may all be indicators of blackout likelihood. It could be argued that these factors make adolescents particularly vulnerable, for example, in a university environment, students are often distanced from parental influence while at the same time they are encouraged to participate in binge-drinking culture [13, 14]. Common among students, both ‘pre-drinking’ (drinking large quantities of alcohol at home before going out with the purpose of getting drunk cheaply) and drinking games (typically involving quickly ingesting large quantities of spirits), are known to increase the chance of experiencing an MBO [3, 15, 16]. Alcohol blackouts can have serious detrimental effects for the individual experiencing them, for example they are associated with a higher risk of personal injury [17], and increased likelihood of engaging in vandalism, physical aggression, and sexual activity with strangers [8]. In addition, studies have shown that around 50% of university students have experienced an MBO within the preceding 12-months [12, 18], highlighting the endemic nature of the MBO event experienced by young adults.

Since alcohol-induced MBOs are endemic in some young adult populations, to what extent do the consequences of this extreme binge-drinking impart any damage to cognition or the brain? We already know that alcohol detrimentally affects plasticity in areas related to memory and learning, thereby altering cognitive processes and normal functioning [19]. We also know that alcohol can cause harm to the developing brain through prenatal exposure [e.g., 20, 21], and during early adolescence [22, 23], thereby changing the developing brain. It is therefore plausible to expect that frequent blackout experiences, constituting extreme binge-drinking episodes, would potentially alter the structure of neural networks. In addition, some pre-existing neuroanatomical differences may be present between individuals who progress into heavy drinking, and therefore regularly experience MBOs, and those who do not [24], suggesting a predisposition towards heavy alcohol drinking. Indeed, longitudinal work by Squeglia and colleagues [25] reported reduced grey matter volume in alcohol-naive adolescents who later transitioned to moderate binge drinking. Subsequent drinking by these individuals resulted in further abnormal reduction in the volume of subcortical and temporal brain structures [25].

Since the brain continues to develop throughout adolescence, with cognitive and structural changes observable even in the mid-20s [26, 27], it is critical to understand whether or not an alcohol-induced MBO imparts any lasting damage to cognitive functioning during young adulthood. There has however been very little investigation of episodic memory in those who regularly experience MBOs. One recent review on alcohol related MBOs reported only two studies which included a test of memory [28]. These papers both showed that alcohol impaired memory for contextual details (i.e., the context surrounding or embedded with a to be remembered item) in participants who experienced blackouts [29, 30]. These findings suggest the possibility that the linking of context with an episodic memory is suppressed by the experience of memory blackouts. More simply, after an alcohol-induced blackout, newly created memories might be less rich in detail.

How does memory operate under the influence of alcohol? A number of studies have investigated episodic memory performance when intoxicated, for example, freely recalling previously studied words, in any order, has been shown to be impaired following low doses of alcohol [31, 32]. Conversely, cued recall, where recall is prompted by the presentation of visually degraded words or word stems for example, is unaffected by the presence of alcohol [31]. Weafer and colleagues [33] showed reduced recall in a cued recall task for emotional stimuli when alcohol was given prior to encoding, in comparison to a placebo control. In fact, alcohol disrupted performance in both cued recall and memory recognition tasks (e.g., do you remember seeing this word before, yes or no?) for emotionally valanced stimuli when alcohol was given prior to encoding, compared to afterwards during consolidation [33]. This result implies that emotionally valanced stimuli may be more deeply encoded than neutral stimuli, and subsequently more affected during intoxicated states; Craik [34] suggests that encoding can be improved by processing items more deeply, i.e., encoding with meaningful analysis. Further, Curran and Hilderbrandt [35] proposed that alcohol may impair encoding of contextual details—peripheral information which could assist in deeper processing. In sum, alcohol appears to significantly impair episodic memory when given prior to encoding, with associated details to the episode being most affected.

Towards our goal of understanding memory performance in the aftermath of an MBO event, we conducted a series of standard episodic memory paradigms on participants who reported experiencing at least 9 MBOs in the preceding 12-months (MBO group). We compared their performance with a control group who have never experienced memory loss as a result of binge-drinking. We employed a free recall task as a baseline for memory retrieval performance, and a serial recall task to assess memory for events in their order of occurrence [36]. We also added a depth of encoding manipulation to an immediate and delayed free recall task which compared recall for items embedded within a sentence context (deep encoding condition) vs. orthographic changes in items (shallow encoding condition). We did this to investigate if recall for items embedded in a context is affected more by an alcohol-induced MBO compared to our shallow encoding manipulation. The delay component (three minutes) within the depth of encoding task was included to assess the impact of frequent MBO events on memory consolidation over time.

Across the three experiments we expected to find that, while sober, performance between both control and MBO groups would be comparable, as observed in previous literature [29, 37]. In line with Wetherill and Fromme [29], and as suggested by Curran & Hildebrandt [35], we hypothesised an increased detriment to recall for items embedded within a context (deep compared to shallow encoding) after ingesting alcohol for our MBO group, compared to controls, in our depth of encoding experiment. The novelty of our studies concerns the subsequent testing of our MBO participants when sober and after experiencing a blackout (<20 hours), to examine if any deficits in memory performance remain. We predicted that our MBO participants would show significant reductions in recall compared to baseline (*before-alcohol*) in all three experiments, indicating a lack of recovery in memory performance after the MBO event.

## Materials and methods

### Design

Participants from the University of Stirling were recruited via online advertisements (no specific exclusion criteria) and asked to complete a general questionnaire examining their alcohol use, behaviours, and familial/peer group relationships with alcohol. Individuals meeting the inclusion criteria for participation in the laboratory-based study then received a follow-up email invitation to take part. These criteria were: (1) either never having experienced an MBO or experiencing 9 or more MBOs in the past 12 months, (2) being aged between 18 and 25 years, and (3) being a fluent English language speaker. In total, 53 participants were recruited, consisting of a control group (*n* = 24, 12 males, mean age = 20.17, *SD* 1.99), and experimental group (MBO group) (*n* = 29, 11 males, mean age = 19.55, *SD* = 1.38). Our control group reported either abstinence from alcohol or drinking alcohol only on very rare occasions, and responses from both groups are given in Table 1. All MBO and control participants in the laboratory experiments were students at the University of Stirling. At the first testing session, participants completed each of the 3 behavioural studies sober, and then repeated the experiments following a scaled dose of alcohol. All participants were compensated for their time with either course credit tokens, or £15. The MBO group were invited to return to the laboratory following a blackout event. On this visit, they completed the 3 behavioural experiments for a third time when sober again, and received additional course credit or money. Of the invited MBO group, 23 participants returned for the additional testing session (10 males, mean age = 19.43, *SD* = 1.2; mean number of days between testing sessions = 24.74 ±30.77). The reduced number of participants at this testing session reflects normal experimental drop-out and is not indicative of drinking-related problems. The studies, and protocol for administration of alcohol, were approved by the NHS, Invasive and Clinical Research committee at the University of Stirling.

**Table 1:** Self-reported frequency of drinking behaviours between MBO (n = 29) and Control (n = 24) groups.

	Group	Never	10 or less	11–20 times	21–30 times	Over 30 times	Chi Square (df)	p value	Fisher exact p values
Drinking sessions, per month	MBO	0	14	10	4	1	16.543 (4)	0.002**	0.0002424**
	Control	3	20	0	1	0			
Drunk instances, per year	MBO	0	1	1	7	20	49.188 (4)	< .001**	6.416E-14**
	Control	7	17	0	0	0			
Binge drinking episodes, per year *	MBO	0	1	2	6	20	45.555 (4)	< .001**	6.416E-14**
	Control	10	12	1	0	0			
		Never	1–4 times	5–8 times	9–12 times	Over 12 times			
Fragmentary MBOs, per year	MBO	0	0	0	14	15	53 (2)	< .001**	1.283E-15**
	Control	24	0	0	0	0			
Enbloc MBOs, per year	MBO	0	13	3	7	6	53 (4)	< .001**	1.283E-15**
	Control	24	0	0	0	0			

Drinking characteristics by frequency of response, and with statistical comparison between groups. Note that chi-square tests for independence may be inappropriate if any expected frequencies are below 5, therefore we also provide Fisher exact p values.

* Defined as more than 6 units of alcohol in a single session.

#### Free and serial recall tasks

All experiments were presented using experimental software E-Prime 1.2 (Psychology Software Tools, Pittsburgh, PA). In both the Free and Serial tasks, participants were presented with 3 blocks of 15 study words on a computer screen and asked to remember them. Stimuli were word lists taken from Roediger and McDermott [38], totalling 270 unique stimuli split into 18 blocks (9 blocks free recall task, 9 blocks serial recall task). Blocks for each individual task were presented pseudo-randomly, counterbalanced across participants. In study blocks, individual words were presented for 1000ms, followed by a blank inter-trial interval of 2000ms. Following each study block of 15 words in the free recall task, participants were asked to recall as many words as they could remember, in any order, by typing their response onto the screen using a keyboard. They were given as much time as they wanted to complete the recall component for each block. The procedure was identical for the serial recall task, except participants were explicitly asked to recall stimuli in the order in which they had been presented.

#### Depth of encoding task

The experiment consisted of 4 blocks of 15 randomly presented words; block order was also randomised, and blocks were split evenly between shallow and deep encoding manipulations. All word stimuli were generated from the MRC Psycholinguistic Database [39, 40] and were 5–9 letters in length, contained 2–4 syllables, and had a familiarity rating of 300–600. A total of 180 stimuli were used in the experiment, split into six blocks of deep and six of shallow stimuli, with the use of each individual block counterbalanced across all participants. In the shallow encoding blocks, stimuli were presented in either lowercase or capital letters for 3000ms. Participants were then asked if the word displayed had been in lowercase letters (yes/no judgement, response counterbalanced between participants, no time limit). In the deep encoding blocks, a sentence with a missing word appeared on-screen for 3000ms, followed by a target word below the sentence for an additional 3000ms. Participants were asked if the target word fitted the sentence (yes/no judgement, response counterbalanced between participants, no time limit to respond). Time taken between each trial for both shallow and deep encoded stimuli was 1000ms. Encoding manipulations (case judgement vs sentence) were based on methods from Craik and Tulving [41]. At test, for both shallow and deep conditions, participants were asked to freely recall as many words as possible, entering responses using a keyboard (immediate recall condition). They had unlimited time to do this. They were then given a distractor task for 3 minutes (Sudoku puzzles), followed by a repeated test session (delayed recall condition).

### Procedure and alcohol protocol

Prior to attending the laboratory, participants were advised of exclusion criteria, and that they would be required to drink alcohol. Exclusion criteria included being under the age of 18, possibility of pregnancy, use of prescribed medication that may interact with alcohol (excluding the contraceptive pill), or previous substance abuse problems. Participants were asked to avoid alcohol for 24-hours and food for between 3 and 4 hours before the study.

Upon arrival, photographic identification, written consent and a breathalyser test (Dräger Alcotest® 3000; Lϋbeck, Germany) were provided by participants. Height and weight were recorded and entered into an alcohol-dose formula [42], along with gender and age. The formula was designed to dose each participant with enough alcohol to reach a Blood Alcohol Content percentage (BAC) of 0.06%, estimated at consistent intervals throughout testing from breath alcohol content (BrAC).

In the first lab visit participants completed all studies when sober, before receiving undiluted 37.5% proof vodka in a glass tumbler with an optional glass straw. Prior to consumption, the vodka was kept in a freezer to minimise taste intensity. Participants were then asked to drink their vodka dose ‘as quickly as was comfortable’ to elicit a rapid spike in BAC. Fifteen minutes after alcohol consumption they gargled with water to remove any residue trace alcohol in the mouth, before being breathalysed. They then repeated the 3 studies, submitting to additional breathalyser tests at regular intervals to measure the BAC spike and decline. In total, participants gave five BrAC recordings during the course of participation. Table 2 details the quantity of alcohol administered, the mean time taken to consume the alcohol, and subsequent mean BrAC readings across the duration of the studies. Participants were asked to remain in the laboratory until their BrAC had dropped below the Scottish driving limit (BrAC 0.22mg/l, BAC 0.05%) during which time they were offered soft drinks.

**Table 2:** Alcohol dose, drinking time, and mean BrAc.

				Breath Alcohol (mg/l)		
	Vodka (ml)	Alcohol (g)	Drink Duration (secs.)	BrAC	Peak BrAC	Final BrAC
Whole Group	94.75 (22.1)	35.533 (8.29)	73.42 (83.76)	0.20 (0.06)	0.37 (0.07)	0.19 (0.04)
Controls (n = 24)	97.75 (22.28)	36.656 (8.35)	72.82 (92.35)	0.22 (0.06)	0.36 (0.08)	0.20 (0.05)
MBO (n = 29)	92.28 (22.03)	34.603 (8.26)	73.83 (79.15)	0.19 (0.05)	0.33 (0.04)	0.18 (0.04)

Means with standard deviations given in brackets.

The studies were presented in two counterbalanced blocks–the free recall and serial recall studies were combined into one block, and the depth of encoding task in another block. The free and serial study word lists were utilised in a DRM recognition memory task which was presented immediately following the serial task. Analysis of this recognition memory task was outside the scope of this manuscript focussing on recall and is therefore not reported. The free recall task always came before the serial recall task, to reduce influence of any memory strategy or heuristic employed in the serial recall task being applied to the free recall task. Presentation order of the two blocks was sequentially changed between participants, and also within participants when on returning visits (MBO group).

#### MBO protocol

The follow-up visit from the MBO group was timed to take place within 20 hours after experiencing an MBO. Participants were asked to keep a drinking diary over the course of six weeks, which consisted of their self-reported alcoholic beverage consumption on each day of the week, for six weeks, beginning at the onset of sign up to the study, used to track their average drinking behaviour. If the participant attended a drinking event which resulted in a blackout, they were asked to contact the researcher to arrange a testing session in the laboratory. To be clear, *no participants* were asked to binge-drink for the primary purpose of this experiment, their follow-up visits were voluntary and at their own instigation. The *after-MBO* studies all took place in the afternoon, with no tests conducted before midday to allow adequate time for the participant to sleep and recover and detoxify (Mean sleep duration = 6.55 hours ±2.05). On arrival all participants were breathalysed and only tested if their BrAC reading was 0.00 mg/l, signifying their return to a sober state. Participants were then asked to complete a consent form, verbally questioned on when they started and stopped drinking, duration and quality of sleep, and details of their blackout experience. All MBO participants confirmed having experienced a memory blackout prior to testing.

We report notable drinking characteristics given by MBO participants who returned for follow-up testing in Table 3. Participant’s self-reported drinking behaviour is also given, recorded from participant’s drinking diaries. One of the male participants’ diary data was not filled in correctly, hence only data from 22 of the 23 are included in those figures. We also report the average number of drinking sessions per week, the amount of alcohol drank in one week, average amount of alcohol drank in any one session, and the participant’s maximum alcohol drank for any one session. Note that these reports are likely to be underestimates due to the fact that reported values entail only what our participants remembered drinking at a particular event [see 43]. Furthermore, UK definitions of binge-drinking suggest 6 or more units in any one session (for females, 8 units for males) constitutes a binge-drinking episode. Data from Table 3 suggests that our MBO participants engage in drinking alcohol 1.89 times per week, yet when they drink they consume more than 6 (or 8) units for each session, i.e., our MBO participants binge-drink heavily.

**Table 3:** Self-reported frequency of drinking behaviours of MBO group.

	Never	10 or less	11–20 times	21–30 times	Over 30 times
Drinking sessions, per month (n = 23)	0	11	7	4	1
Drunk Instances, per year (n = 23)	0	1	4	5	16
Binge-drinking episodes, per year * (n = 23)	0	1	2	4	16
	Never	1–4 times	5–8 times	9–12 times	Over 12 times
Fragmentary MBOs, per year (n = 23)	0	0	0	12	11
Enbloc MBOs, per year (n = 23)	0	11	3	5	4
	UK Units		Grams Ethanol (g)		Number of Sessions
Per week (n = 22)	26.99 (11.404)		215.918 (91.235)		1.89 (0.661)
Per session (n = 22)	13.364 (4.342)		106.912 (34.739)		
Max per session (n = 22)	21.225 (8.326)		169.8 (66.611)		

Frequency of responses to drinking behaviour questions, and quantity of alcohol consumed over a 6-week period given as mean scores with standard deviation in brackets. A drinking session refers to a single drinking event of unspecified duration.

* Defined as more than 6 units of alcohol in a single session.

### Statistical analysis

**Fig 1:**
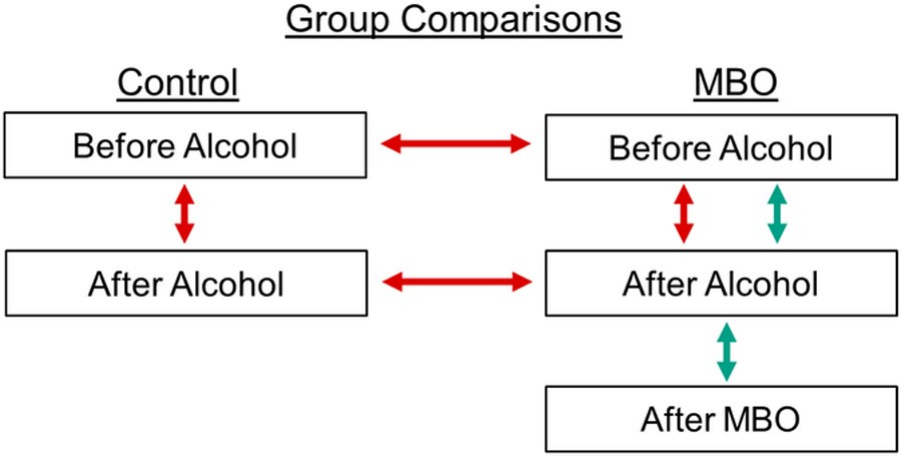
Analysis structure. Displays the design structure for all three experiments. Red arrows show the between group comparisons, comparing control and MBO participants before and after-alcohol. The green arrows highlight the design for the analysis of the MBO group data only.

We used linear mixed models (LMM) to analyse data from all experiments and to account for the difference in sample size between control and MBO participants, and multiple samples taken from the same participants at different timepoints (see S1 and S2 Appendices for full model outputs and structure). In the free and serial recall tasks we assessed the percentage of accurately recalled words, and frequency of false alarms, with fixed effects of alcohol (before and after alcohol), and group (control and MBO). We also did this for the MBO group only, looking at the impact of MBOs, compared to before and after drinking alcohol conditions (see Fig 1). To be clear, when we discuss an after-MBO effect, or a blackout effect, we are referring to any statistical difference between sober (*before-alcohol*) and *after-MBO* conditions. We used Bonferroni corrected paired t-tests, reporting Bonferroni adjusted p values, to compare the within-group means for the MBO group. In addition, in the serial recall task we further investigated sequence length (recalling 2 or more words in the correct order).

For the depth of encoding task, models were conducted on accurately recalled words (%) and false alarms split by alcohol conditions, both between groups and within the MBO group only. We analysed fixed effects of group, alcohol, depth (shallow vs. deep) and delay (immediate vs. 3-minute delay), and also the interactions between these effects. All analysis was conducted using R [44] and *nlme* [45]. Only results of interest are reported (all significant effects and selected non-significant results), and the effect sizes of planned contrasts, given by *r*_*contrast*_ *= $\sqrt{\frac{t^{2}}{t^{2}+df}}$* [46, 47].

Since we were interested in whether individual participants were significantly impaired after experiencing an MBO, we first ruled out the possibility that sleep impacted performance on the tasks, by correlating time slept with the difference in recall performance between *before-alcohol* and *after-MBO* conditions. Because we are interested in support for the null hypothesis, we include equivalent Bayes Factors (*K*) for all tests conducted [48]. Finally, to further quantify the differences between *before-alcohol* and *after-MBO* conditions in individuals, we resampled the ordering of *before-alcohol* and *after-MBO* conditions for each MBO participant 2000 times to build test distributions of possible mean differences (converted to z scores) between *before-alcohol* and *after-MBO* conditions (see Fig 1 for comparisons). For all three tasks we compared each individual participant’s sampled mean difference (z scores) between *before-alcohol* and *after-MBO* conditions to our resampled test distributions to verify precisely how many participants showed significant memory deficits in each task.

## Results

### Between groups analysis: Control vs. MBO participants

#### Free recall

**Fig 2:**
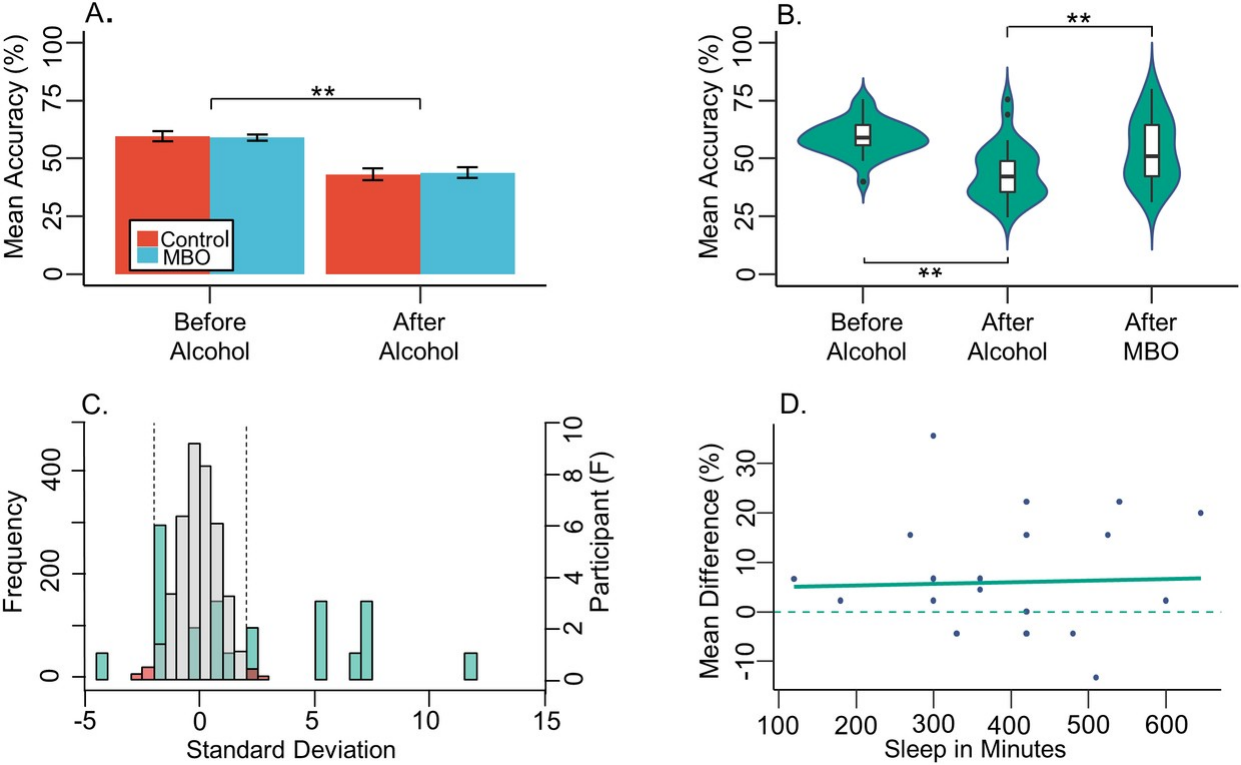
Free recall. (**A**) bar graph shows between control and MBO group mean accuracy (%) of freely recalled words, both before and after ingesting alcohol. Error bars depict standard mean error with ** denoting significance at p < .01, and * < .05. (**B**) violin plots displaying the distribution of MBO participant responses, with embedded box and whisker plots across all three test conditions (*before-alcohol*, *after-alcohol*, *after-MBO*). Outliers appear as dots above or below the box and whisker plots. Note the change in spread of the distribution for the *after-MBO* condition in comparison to before and *after-alcohol* conditions. (**C**) histograms depicting the resampling analysis for the free recall task in the MBO group. The left y axis shows the frequency of resampled mean differences, converted into z-scores, between *before-alcohol* minus *after-MBO* conditions. Bar width is 0.5 standard deviations. Grey bars depict roughly 95% of the resampled distribution, and the red bars show the 2.5% tails at either side, demarcated by vertical dashed lines. Overlaid green bars are a separate histogram (right y axis) showing the frequency of participants’ mean differences (z-scores), with the same bar width of 0.5 standard deviations. Therefore, the figure displays how many participants are significantly different from our resampled distribution of mean differences, as these participants would be outside of the grey area on the resampled histogram. (**D**) scatterplot displays the difference between the mean accuracy (%) for freely recalled words *before-alcohol* minus *after-MBO*, correlated with reported minutes slept, within the MBO group.

Comparing the free recall accuracy between groups, we found a significant main effect of alcohol, *X*^*2*^(1) = 63.96, *p*< .0001. To summarise the model, the main effect of alcohol was a reduction *after-alcohol* in mean accuracy for both groups compared to *before-alcohol*, *b* = -7.875, *t*(52) = -10.98, *p* < .0001, *r* = .84 (see Fig 2A). No main effect of group was present, *p =* .*967*, nor did the factors of group and condition interact, *p =* .*637*.

#### Serial recall

**Fig 3:**
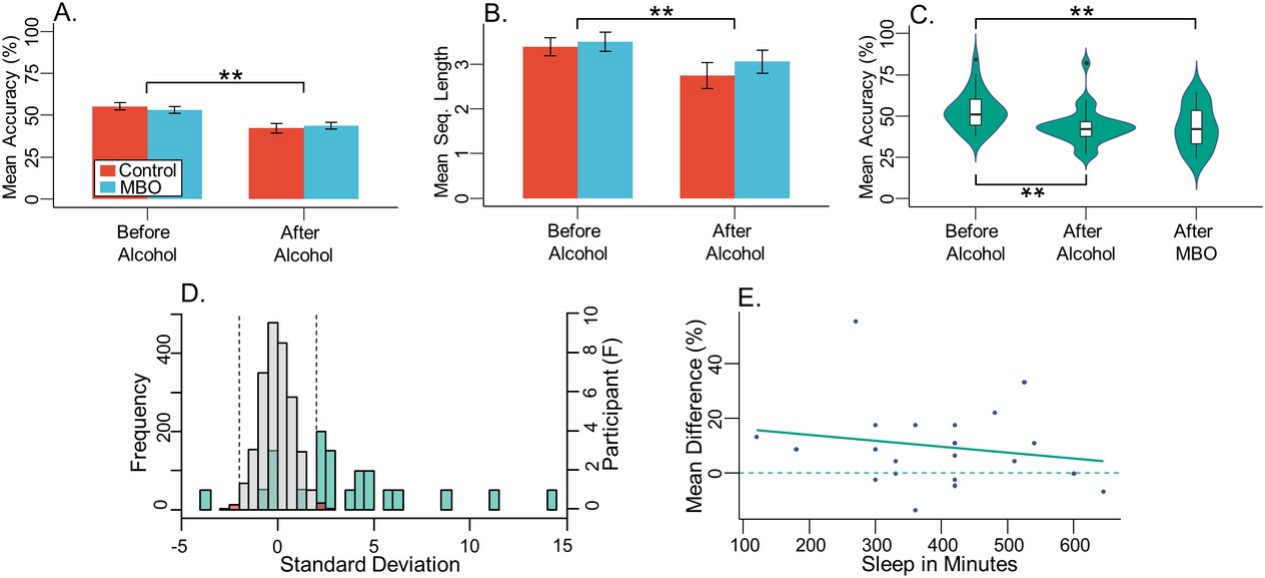
Serial recall. (**A**) bar graph shows between control and MBO group mean accuracy (%) of serial recalled words, both before and after ingesting alcohol. Error bars depict standard error of the mean, with ** denoting significance at p < .01, and * < .05. (**B**) shows the same as (**A**), except that mean accuracy (%) for sequence length, i.e., the average number of words recalled in sequence, is plotted. (**C**) violin plots show the distribution of MBO participant responses in the serial recall task for overall recall accuracy, with embedded box and whisker plots across all three test conditions (*before-alcohol*, *after-alcohol*, *after-MBO*). Outliers appear as dots above or below the box and whisker plots. (**D**) histograms depicting the resampling analysis for mean accuracy % in the serial recall task for the MBO group. The left y axis shows the frequency of resampled mean differences, converted into z-scores, between *before-alcohol* minus *after-MBO* conditions. Bar width is 0.5 standard deviations. Grey bars depict roughly 95% of the resampled distribution, and the red bars show the 2.5% tails at either side, demarcated by vertical dashed lines. Overlaid green bars are a separate histogram (right y axis) showing the frequency of participants’ mean differences (z-scores), with the same bar width of 0.5 standard deviations. (**E**) scatterplot displays the difference between the mean accuracy (%) for serial recalled words *before-alcohol* minus *after-MBO*, correlated with reported minutes slept, within the MBO group.

Groups did not differ in the mean accuracy of recall within the serial recall task, however there was a significant main effect of alcohol, *X*^*2*^(1) = 42.08, *p* < .0001 (see Fig 3A). Like the free recall task, alcohol reduced recall similarly for both groups compared to *before-alcohol*, *b* = -5.53, *t*(52) = -7.9, *p* < .0001, *r* = .74. When analysing the mean number of words recalled in sequence, we again found a main effect of alcohol, *X*^*2*^(1) = 7.2, *p* = .007 (see Fig 3B). After drinking alcohol, the sequence length was significantly reduced for both control and MBO groups, *b* = -0.27, *t*(52) = -2.74, *p* = .009, *r* = .36.

#### Depth of encoding

**Fig 4:**
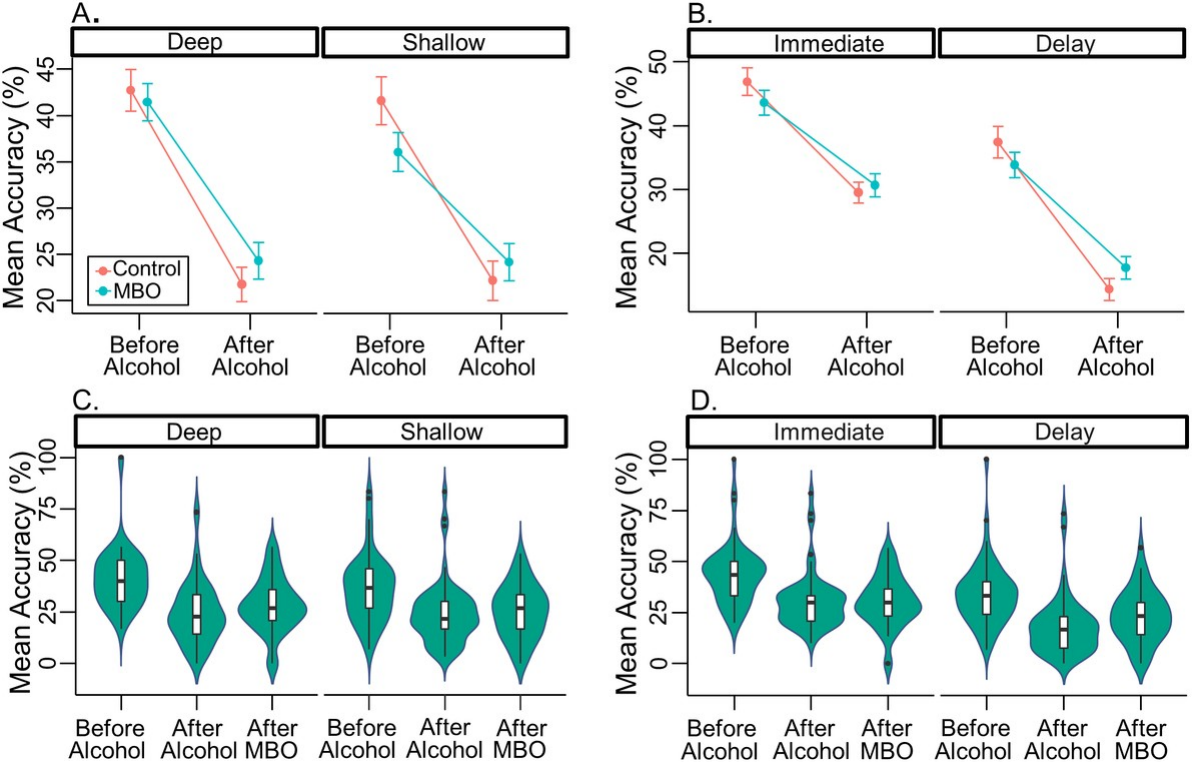
Depth of encoding accuracy. (**A, B**) line graphs showing between control and MBO group mean accuracy (%) for freely recalled words in the depth of encoding task, both before and after ingesting alcohol. (**A**) displays data for deep and shallow conditions collapsed across delay, whereas (**B**) shows the differences between immediate and delayed recall conditions, collapsed across deep and shallow. Error bars depict standard error of the mean. (**C, D**) violin plots show the distribution of MBO participant responses in the depth of encoding task for the shallow vs deep (**C**), and immediate vs delay recall (**D**) conditions, with embedded box and whisker plots across all three test conditions (*before-alcohol*, *after-alcohol*, *after-MBO*). Outliers appear as dots above or below the box and whisker plots.

For the depth of encoding task, our LMM analysis on the total number of words recalled (%) between groups highlighted a significant interaction effect between the alcohol (before vs after) and delay (immediate vs. 3 minute delay) conditions, and also alcohol and depth (shallow vs. deep), *X*^*2*^(1) = 6.32, *p* = .0119. To summarise the model, overall accuracy reduced after drinking alcohol, *b* = -8.68, *t*(51) = -12.482, *p* < .0001, *r* = .868. The 3 minute delay (with distraction task) also reduced performance compared to immediate recall, *b* = -5.911, *t*(103) = -16.444, *p* < .0001, *r* = .851. We also found that fewer words were recalled in shallow than in deep conditions, *b* = .779, *t*(209) = 2.167, *p* = .0314, *r* = .148. Further, we found an interaction between group and alcohol, such that alcohol had less of an effect on the MBO group than the control group, *b* = 1.424, *t*(51) = 2.048, *p* = 0.0457, *r* = .276; although the MBO group recalled fewer words to begin with, the control group showed a larger reduction in percentage words recalled *after-alcohol* (see Fig 4A and 4B). Drinking alcohol also interacted with the delay in test, *b* = -1.077, *t(*103) = -3.009, *p =* .0033, *r* = .284; there was a larger drop in recalled words following both the delay and after drinking alcohol compared to the reduction in accuracy *after-alcohol* but immediate recall (see Fig 4B). Finally, irrespective of group, *before-alcohol* more words were recalled in the deep than in the shallow condition, yet *after-alcohol* no differences were found between shallow and deep encoding conditions, *b* = -0.893, *t*(209) = -2.497, *p* = 0.013, *r* = 0.17. To briefly summarise, alcohol reduced recall most for deep encoded conditions, and the drop in recall was largest for the control group.

In summary, alcohol impaired both groups of participants in free and serial recall tasks to a similar extent. In contrast, behavioural performance between groups differed in the depth of encoding task where control participants exhibited greater reduction in recall accuracy after alcohol than the MBO group.

### Within MBO group analysis

#### Free recall

For the MBO group only, drinking alcohol significantly impaired recall, *X*^*2*^(2) = 33.79, *p* < .0001. Specifically, *after-alcohol* there was a significant drop in recall compared to *baseline* (*before-alcohol*) performance (*p* < .0001), yet after experiencing an MBO (*after-MBO*) we observed an improvement in recall performance compared to the *after-alcohol* condition (*p* = .012; see Fig 2B). There was no difference between the two sober conditions (*before-alcohol* and *after-MBO*) (*p* = .068). Furthermore, we found no difference in false alarms within the MBO group, or between alcohol conditions.

#### Serial recall

Within the MBO group there was a main effect of alcohol on total words recalled, *X*^*2*^(2) = 25.92, *p* < .0001 (see Fig 3C). Bonferroni corrected pairwise comparisons revealed that alcohol significantly reduced recall compared to *before-alcohol*, *p* < .0001. *After-MBO* recall remained reduced compared to *baseline*, *p* = .0007. Unlike the free recall task, recall between *after-alcohol* and the *after-MBO* conditions did not differ, *p* = 1, suggesting no recovery in recall after a blackout. There was no effect of alcohol on sequence length, suggesting that the average length of sequences held in memory was not affected by alcohol, only total recall, and therefore number of sequences. In addition, there was no significant difference in false alarm rates within the MBO group.

#### Depth of encoding

The LMM model which best fitted the MBO group data showed an interaction between the factors of alcohol (*Before-alcohol*, *after-alcohol*, *after-MBO*) and depth (shallow vs. deep), *X*^*2*^(2) = 7.321, *p* = .0257. We found that recall was reduced compared to *before-alcohol* both *after-alcohol* (*p <* .0001) and *after-MBO* (*p <* .0001), with no difference between recall *after-alcohol* vs. *after-MBO* (*p = 1*). Overall, fewer words were recalled in the shallow condition than in the deep condition, *b* = -1.438, *t*(159) = -3.44, *p* = 0.0007, *r* = .263. Also, recall was reduced for delayed conditions compared to immediate recall, *b* = -5.032, *t*(78) = -12.038, *p* < .0001, *r* = .806. An interaction between alcohol and delay, *b* = 1.288, *t*(78) = 2.096, *p* = .0393, *r* = .231, demonstrated that *after-alcohol* and *after-MBO* recall was similar for immediate recall conditions, but delayed recall conditions showed an improvement in performance *after-MBO* relative to *after-alcohol* (see Fig 4C and 4D). A final interaction between alcohol and depth revealed that *after-alcohol*, there was a drop in both shallow and deep encoding conditions, however this was greater for deeply encoded words, *b* = -1.263, *t*(159) = -2.181, *p* = .0307, *r* = .17. There was a small increase in recall of deeply encoded words *after-MBO* compared to *after-alcohol*, however no recovery in the recall of shallow encoded words.

In sum, we found evidence for reduced performance *after-MBO* compared to *before-alcohol* in our MBO group in two of the three tasks (serial recall and depth of encoding tasks).

#### Individual analysis of MBO effects

**Fig 5:**
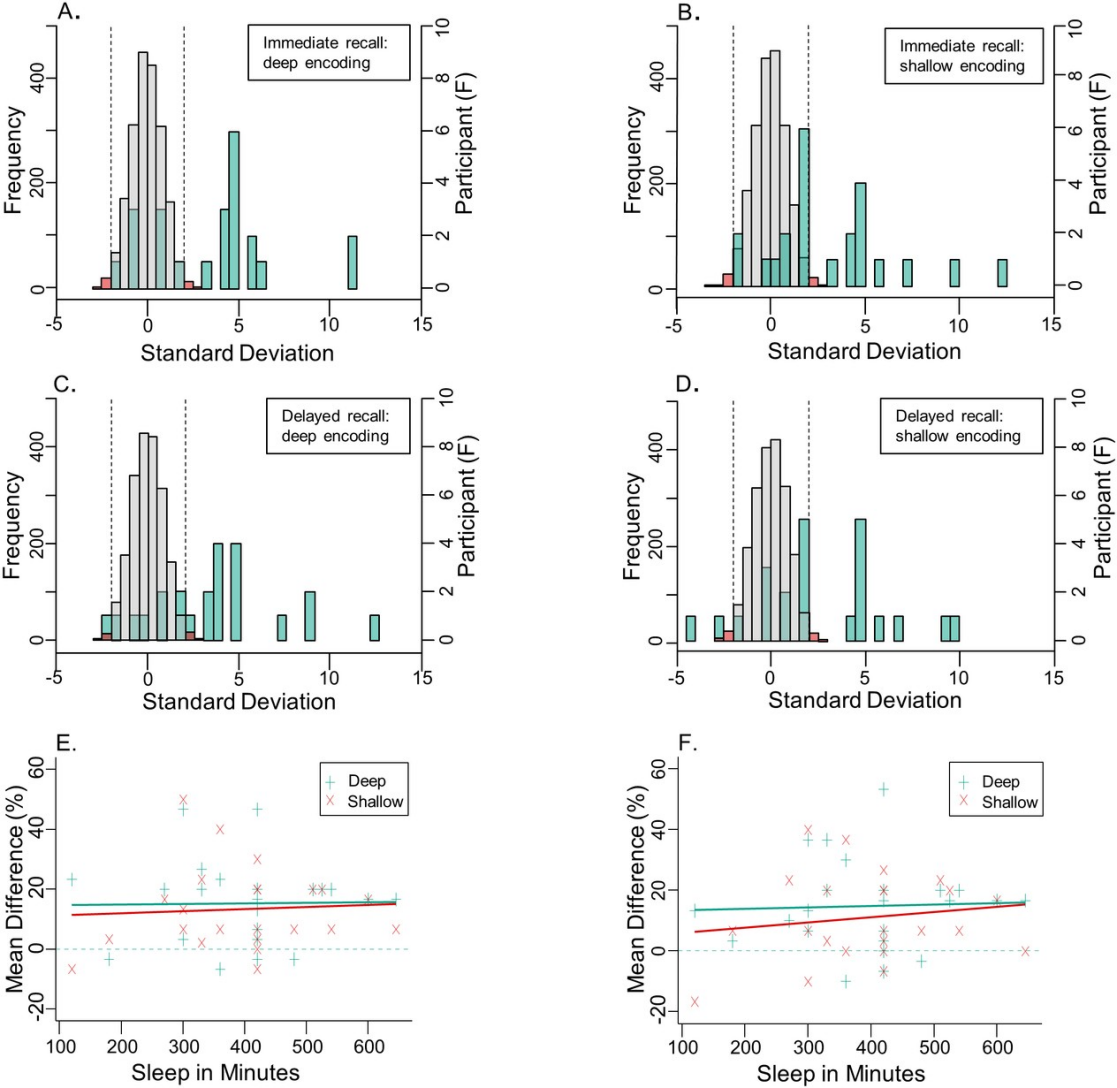
Depth of encoding resampling and sleep data. Histograms (**A**, **B**, **C**, & **D**) depict the resampling analysis for the depth of encoding task in the MBO group. The left y axis’ show the frequency of resampled mean differences, converted into z-scores, between *before-alcohol* minus *after-MBO* conditions for the immediate recall, deep encoding (**A**), immediate recall, shallow encoding (**B**), delayed recall, deep encoding (**C**), and delayed recall, shallow encoding (**D**) conditions. In all panels bar width is 0.5 standard deviations. Grey bars depict roughly 95% of the resampled distribution, and the red bars show the 2.5% tails at either side, demarcated by vertical dashed lines. Overlaid green bars are a separate histogram (right y axis) showing the frequency of participants’ mean differences (z-scores), with the same bar width of 0.5 standard deviations. (**E**) scatterplot displays the difference between the mean accuracy (%) for immediately recalled words in the depth of encoding task, *before-alcohol* minus *after-MBO*, correlated with reported minutes slept, within the MBO group. (**F**) shows the same as (**E**), except for the delayed recall conditions.

First of all, we investigated whether any blackout effects in any task within the MBO group could be attributable to a lack of sleep. No relationship between sleep quantity and performance after blackout was found for free recall (*p* = .876; adjusted R^2^ = -0.046; K = 0.382; see Fig 2D), serial recall (ACC: *p* = .394; adjusted R^2^ = -.011; K = 0.498; See Fig 3E; Sequence Length: *p* = .322; adjusted R^2^ = .001; K = 0.548), or depth of encoding conditions. In more detail, immediate recall accuracy was not correlated with sleep, for either deep (*p* = .933, adjusted R^2^ = -0.933, K = 0.38) or shallow (*p* = .777, adjusted R^2^ = -0.044, K = 0.39) encoding measures. Likewise, delayed recall accuracy was unaffected (deep: *p* = .865, adjusted R^2^ = -0.046, K = 0.383; shallow: *p* = .495, adjusted R^2^ = -0.024, K = 0.451) (see Fig 5E and 5F). Taken together, these results suggest weak evidence favouring the null hypothesis [49] and thus that individual blackout effects in any of the tasks may not be due to a lack of sleep.

In addition, we ran resampling analyses for each individual’s performance between *before-alcohol* and *after-MBO* conditions in all the tasks to quantify the significance of blackout effects. For the free recall task, accuracy in 10 out of 23 participants (43.5%) was significantly impaired after experiencing an MBO (see Fig 2C). Twelve participants (52.2%) showed no difference between *before-alcohol* and *after-MBO* conditions, whereas 1 participant (4.3%) significantly improved after blackout. During the serial recall task, 17 out of 23 participants (73.9%) had significantly poorer recall accuracy *after-MBO* (see Fig 3D). Five participants (21.7%) showed no difference between *before-alcohol* and *after-MBO* conditions, whereas 1 participant (4.3%) significantly improved *after-MBO*. Finally, in the depth of encoding task, (see Fig 5A–5D), for deeply encoded items at immediate recall, 15 (65.2%) showed significant differences between *before-alcohol* and *after-MBO* conditions, while 8 (34.8%) showed no effect. For deeply encoded items with delayed recall, 15 (65.2%) showed a significant *after-MBO* impairment, while 7 (30.4%) showed no effect and 1 (4.3%) an improvement after blackout. In the shallow encoding, immediate recall condition, 11 participants (47.8%) showed the effect and 12 (52.2%) did not. In the shallow encoding, delayed recall condition, 10 participants (43.5%) showed the effect, 11 (47.8%) did not, and 2 participants (8.7%) improved *after-MBO*. These results suggest that the deeply encoded conditions were most affected by binge-drinking until blackout.

## Discussion

We aimed to examine whether young adults who experience a high volume of MBOs are poorer in terms of episodic memory performance compared to non-blackout controls, either when sober or after ingesting alcohol. Specifically, we hypothesised in line with other literature [29, 30] that our MBO participants would be most affected by the presence of alcohol when items would be presented in a context (sentence context, depth of encoding task). Against our hypothesis, we found that control participants showed increased recall when sober, and subsequently a larger fall in performance, compared to MBO participants after ingesting alcohol on the depth of encoding task. No significant differences between control and MBO participants were found when sober, or after ingesting alcohol, on free and serial recall tasks.

We further aimed to determine whether an alcohol-induced MBO leads to impaired recall the next day which remains beyond the point of recovered sobriety. Examining individuals after an MBO we found delayed recovery of memory (i.e., performance not returning to baseline levels) in serial recall and depth of encoding tasks, and variable recovery in the free recall task. Concerning the free recall task, group level statistics indicated no difference between *before-alcohol* and *after-MBO* conditions, however the data is variable and 43.5% of participants exhibited significantly poorer recall *after-MBO*. No evidence was found to suggest these blackout effects were impacted by a lack of sleep, in fact evidence from Bayes Factor Analysis favoured the null hypothesis that a lack of sleep had no effect on recall performance *after-MBO*. Taken together, these findings suggest that, even when sober, alcohol-induced blackout episodes impart some lasting damage on memory processes.

In our free recall experiment, both groups showed similar recall accuracy when sober and after drinking alcohol, where the amount of words recalled decreased at the same rate. It is known that alcohol spares short term memory in alcoholics, with spans of up to five minutes being reported as unaffected [50, 51], therefore it is unsurprising that there would be few perceivable behavioural differences in immediate recall between groups, particularly at lab-appropriate levels of BrAC. Additionally, within the MBO group, recall *after-MBO* was variable across the group, with 10 participants showing a deficit in relation to sober conditions, while 12 showed no deficit. This pattern of variability may suggest a weak effect size within the overall population of individuals who blackout frequently for free recall, and mirrors findings across studies of hangovers in social drinkers. Some studies have shown no deficit in memory performance [see, for example, 52, 53], but others have found impaired performance during hangovers in free recall tasks [54, 55]. Possibly the differences between findings reflects the design of experiments, either measuring in the laboratory or relying on self-reported drinking behaviour. It is probable that participants drink more in naturalistic studies, like the present investigation, than in lab-based experiments, leading to the increased performance deficits observed in naturalistic studies. Note that a naturalistic design will also lead to variable reporting of MBO effects in the literature, due to the variability in each participant sampled. Time of testing after experiencing an MBO may also serve to weaken any *after-MBO* effects, i.e., differences between baselines and after experiencing a blackout. In the present data sets, we tested all participants within 20 hours of experiencing an MBO, in an attempt to capture alcohol-induced MBO deficits before full recovery. However, the precise time when a blackout occurred is not possible to determine from participant self-report, nor did we examine the rate of recovery after blackout—our studies focussed on finding if any deficit was present after experiencing a blackout.

In comparison to the free recall task, the serial recall task increased cognitive load by asking participants to immediately recall words in the order of their presentation. We found again that alcohol impaired both the number of words recalled, and the length of sequences recalled, in both groups. Although we analysed total words recalled rather than considering just the number of words recalled in serial order, the additional load of trying to remember words in sequence appeared to disrupt recall regardless, in line with previous studies highlighting the effects of cognitive load on attention and memory [56]. In contrast to the free recall task, the MBO group displayed significantly reduced performance on the task after experiencing an MBO, similar to after ingesting alcohol. 73.9% of individuals exhibited consistently poor recall after experiencing an MBO, highlighting the severity of an alcohol-induced MBO on memory performance under demanding task constraints.

In the depth of encoding study, control participants showed a greater drop in performance after alcohol, suggesting that they were more impaired by the presence of alcohol than the MBO group in both immediate and delayed recall. The depth manipulation presented target words in a contextual sentence, or narrative, while the shallow presentation simply asked for a visual recognition judgment (upper- or lower-case letters). After alcohol, both groups performed similarly in deep and shallow conditions however, before alcohol, more words were recalled from the deep context than the shallow. Alcohol is known to affect encoding [57] therefore some may consider a greater drop in performance for deeply encoded items, compared to shallow, following alcohol consumption to be surprising. It may be that deeply encoded items, said to have a stronger memory trace [58], would be more impervious to the effects of alcohol on free recall. However, it is also to be expected that at baseline deeply encoded items are recalled with greater frequency than shallow items, and therefore performance in this condition can fall further than the shallow condition after alcohol, as seems to be the case in the present experiment.

Moreover, our deeply encoded items were presented within a sentence context, which we did not test memory for. It is possible that the decay of memory for this sentence narrative could underlie the drop in performance for deeply encoded items, however, memory for contextual information, such as the sentence narrative in this present experiment, is not necessarily dependent on item recall (for discussions of source memory, see [57, 59–61]. Essentially, our deep (and shallow) encoded items could be said to contain source information which we tested at encoding but did not test at recall. Contextual details for events (what, where, when, etc.) are bound together with the event itself to create an episodic memory, and these contextual (source) details are hypothesised to aid recollection [60]. Alcohol is thought to impair this process; indeed, the loss of some contextual details, such as serial ordering of events, is thought to contribute in part to the experience of a fragmentary MBO [37]. Despite the fact we could not measure source recollection, it is conceivable that recall performance for deeply encoded items would drop to a similar level seen for shallow encoding, after ingesting alcohol.

In addition, our participants showed little overall difference between *after-alcohol* and *after-MBO* conditions in the depth of encoding experiment in terms of the number of words recalled. There was a small recovery in recall *after-MBO* for deep but not shallow encoded words, and for delayed but not immediately recalled words. This statistical “recovery” in delayed recall is not surprising as alcohol consumption reduced memory further for delayed than immediately recalled words, yet note that performance in delayed recall was always worse than immediate recall. Recovery in this context does not suggest that memory is operating as normal again for certain conditions such as deep encoding after the blackout event, it is minor, and relative to the impact of alcohol consumption on memory. Given that previous studies suggest that alcohol impairs encoding of contextual details [35] we speculate that alcohol-induced MBOs also affect encoding of associated details that support recollection, e.g., position in sequence of a word during the serial recall task or sentence narratives for deeply encoded items.

It is important to note that the variability in the *after-MBO* effects found across the three experiments can be explained by task demand differences and the additional cognitive processes these tasks engage in relation to free recall. For example, both our serial recall, and depth of encoding task are more cognitively demanding than simple free recall, involving an ordering of remembered episodes and also a delay to recall. Notwithstanding this, our findings in the three recall tasks are broadly in agreement with the small number of reported MBO studies [29, 37]. Neither Wetherill and Fromme [29], nor Hartzler and Fromme [37], found differences between control and blackout participants before alcohol in immediate recall tasks and across differing paradigms. Similar to our findings, Hartzler and Fromme [37] also found no group differences following alcohol for immediate recall. In contrast to our results, both papers did report an increase in deficit after consuming alcohol for their blackout participants, specifically in delayed recall of narrative details. Although these results after ingesting alcohol were not replicated here, we did not use narrative recall tasks, nor did we administer such a high dose of alcohol to participants as the above-mentioned studies.

Our data for the high-volume blackout group relies on our participant’s self-reporting of their own memory blackout behaviour. We acknowledge that in a naturalistic examination of blackouts it is not possible to identify the strength of the blackout, which introduces a measure of variability into results. Our investigation focussed on instances of extreme binge-drinking leading to MBOs, and whether they impact memory the day afterwards, yet it is important to highlight that blackout effects presented here may be influenced by the presence of hangover symptoms in our participants. Hangover symptoms have also been shown to negatively impact memory [53]. However, note that hangovers and memory blackouts are not mutually inclusive; a blackout can occur with minor or no hangover symptoms, and similarly a hangover can occur without having also experienced a blackout. We have not found any work in the literature that has investigated both hangovers and MBOs concurrently. Critically, while a hangover can present with a multitude of physical symptoms, the experience of those symptoms is subjective. Van de Loo et al. [62] show that the most important determinant of hangover severity is a participant’s own perceived levels of alcohol intoxication. It is important in the future to dissociate the study of hangovers and MBOs to determine the relative impact of both experiences on cognition. It is likely that both experiences impact memory performance when sober, but it is currently unknown whether this is caused by the multitude of physical symptoms experienced during hangover (e.g., nausea, malaise, fatigue, etc.) or an enduring impact of the blackout (caused by alcohol) on hippocampal functioning.

Furthermore, Verster [53] has suggested that a lack of sleep and detectable alcohol BAC% levels at time of testing could explain the mixed results in the literature [for example, 52, 54, 55], since a lack of sleep may inflate the strength of after binge-drinking effects on cognition. We highlight these issues here, and note that we attempted to control where possible for average alcohol intake for our high volume MBO participants, and their estimated time slept after an MBO. All participants reported sleeping, all were tested when sober, and testing took place later in the day allowing time for detoxification. There were no correlations found between sleep and recall accuracy, in contrast we found weak evidence in support of the null hypothesis in all tests conducted. More importantly, we still observed performance deficits in the *after-MBO* condition. Alcohol is suggested to impact sleep quality [43, 63], however, it is worth noting that measures of sleep quality are subjective, include qualitative components [see 43, 64], and by their very nature are likely to strongly correlate with sleep quantity. Future work may focus on quantitative measures of sleep quality affected by alcohol.

We originally hypothesised that people who experience a high volume of MBOs may perform differently in recall tasks compared to people who have never experienced an alcohol-related memory blackout. Our data suggests that in general they do not perform differently, however, a lack of differences between controls and high frequency MBO participants here does not necessarily imply that the two groups of participants are equal. There is a paucity of neuroimaging work examining the impacts of memory blackouts, however, Squeglia et al. [25] examined structural changes in the brains of low-moderate frequency binge drinkers, and highlighted reduced grey matter volume in young adults compared to controls. Similarly, reduced event-related potential (ERP) amplitudes and delay of onset of early onsetting ERP components (e.g., P1, N2, P300, P3b) have been observed in basic cognitive tasks in heavy binge drinkers [e.g. 65, 66]. In a meta-analysis of the binge-drinking literature, Lees and colleagues [67] suggest that abnormal or delayed developmental of pre-frontal regions of the brain may be a consequence of binge-drinking in young adulthood, predisposing people to further alcohol-related harm. While caution is required when making assumptions about whether possible biomarkers would also be apparent within our blackout group during neuroimaging, our young adult participants displayed extreme binge-drinking behaviours showing behavioural deficits in memory after a single acute episode. It is reasonable to propose further examination of these performance differences using neuroimaging methods would constitute a more sensitive test of our hypothesis.

To conclude, the three experiments presented here examined episodic memory performance in people who experience alcohol-related memory blackouts. To the best of our knowledge, this is the first paper to compare frequent blackout participants when sober, after alcohol, and after blackout, and further, contrast their performance with a control group before and after alcohol. We hypothesised that in comparison to controls, MBO participants may show greater deficits in memory performance after drinking alcohol yet found limited group differences before and after alcohol. However, we show that after experiencing a blackout, deficits remained in all three experiments to varying degrees (individual participant data), and group data highlighted significant *after-MBO* effects in the serial recall and depth of encoding tasks. It remains possible that behavioural performance masks underlying differences in cognitive strategies between controls and frequent blackout participants observed in studies of binge-drinking [68, 69]. In sum, our data highlight a deficit in episodic memory performance after experiencing an alcohol-induced memory blackout, that does not correlate with time spent sleeping, and endures beyond the presence of alcohol in the body.

## Supporting information

S1 Appendix Between group analysis model output tables. (DOCX)

S2 Appendix Within MBO group analysis model output tables. (DOCX)
