# Supplementary material for: The morning after the night before: Alcohol-induced blackouts impair next day recall in sober young adults
Source: PLoS One. 2021 May 3;16(5):e0250827. doi: 10.1371/journal.pone.0250827 (PMC8092761; doi:10.1371/journal.pone.0250827)
Supplement: S1 Appendix — (DOCX) [file pone.0250827.s001.docx]

**S1 Appendix. Between Group Analysis Model Output Tables**

***Table S1:1 Free Recall***

| **Model specification** | **Model name** | **Nested Model** | **Fixed Effects** | **Model Comparison** | **Model fit** | | | | | |
| --- | --- | --- | --- | --- | --- | --- | --- | --- | --- | --- |
|  |  |  |  |  | **df** | **AIC** | **BIC** | **LogLik** | **L. Ratio** | **p-value** |
|  | | | | | | | | | | |
| Mean Accuracy (%) | Baseline |  |  |  | 5 | 856.101 | 869.4182 | -423.0505 |  | |
|  | freel1 | Baseline | + Group | free1 to Baseline | 6 | 858.0992 | 874.0799 | -423.0496 | 0.00175 | 0.9666 |
|  | free2 | free1 | + Condition | free2 to free1 | 7 | 796.1364 | 814.7804 | -391.0682 | 63.9629 | <.0001 |
|  | free3 | free2 | + Group:Condition | free3 to free2 | 8 | 797.9142 | 819.2217 | -390.9571 | 0.22212 | 0.6374 |
| free2 model equation: ***MeanACC ~ Group + Condition, random = ~1\|Participant/Group/Condition*** | | | | |  |  |  |  |  |  |
| **Model Summary** | **df** | **Beta** | **SE** | **t-value** | **p-value** |  |  |  |  |  |
| Intercept | 52 | 51.35 | 1.27 | 40.29 | 0 |  |  |  |  |  |
| Group | 51 | 0.05 | 1.27 | 0.042 | 0.9667 |  |  |  |  |  |
| Condition | 52 | -7.87 | 0.72 | -10.976 | 0 |  |  |  |  |  |

***Table S1:2a Serial Recall – Mean Accuracy***

| **Model specification** | **Model name** | **Nested Model** | **Fixed Effects** | **Model Comparison** | **Model fit** | | | | | |
| --- | --- | --- | --- | --- | --- | --- | --- | --- | --- | --- |
|  |  |  |  |  | **df** | **AIC** | **BIC** | **LogLik** | **L. Ratio** | **p-value** |
|  | | | | | | | | | | |
| Mean Accuracy (%) | Baseline |  |  |  | 5 | 845.1995 | 858.5167 | -417.5997 |  | |
|  | serial1 | Baseline | + Group | serial1 to Baseline | 6 | 847.1822 | 863.1629 | -417.5911 | 0.01728 | 0.8954 |
|  | serial2 | serial1 | + Condition | serial2 to serial1 | 7 | 807.1052 | 825.7493 | -396.5526 | 42.07703 | <.0001 |
|  | serial3 | serial2 | + Group:Condition | serial3 to serial2 | 8 | 807.6785 | 828.9861 | -395.8393 | 1.42665 | 0.2323 |
| serial2 equation: ***MeanACC ~ Group + Condition, random = ~1\|Participant/Group/Condition*** | | | | |  |  |  |  |  |  |
| **Model Summary** | **df** | **Beta** | **SE** | **t-value** | **p-value** |  |  |  |  |  |
| Intercept | 52 | 48.65501 | 1.4475981 | 33.61086 | 0 |  |  |  |  |  |
| Group | 51 | -0.18758 | 1.4475981 | -0.12958 | 0.8974 |  |  |  |  |  |
| Condition | 52 | -5.53459 | 0.7005305 | -7.90057 | 0 |  |  |  |  |  |

***Table S1:2b Serial Recall – Mean Sequence Length***

| **Model specification** | **Model name** | **Nested Model** | **Fixed Effects** | **Model Comparison** | **Model fit** | | | | | |
| --- | --- | --- | --- | --- | --- | --- | --- | --- | --- | --- |
|  |  |  |  |  | **df** | **AIC** | **BIC** | **LogLik** | **L. Ratio** | **p-value** |
|  | | | | | | | | | | |
| Mean Sequence Length (%) | Baseline |  |  |  | 5 | 355.6559 | 368.9731 | -172.8279 |  | |
|  | serialseq1 | Baseline | + Group | serialseq1 to Baseline | 6 | 357.084 | 373.0646 | -172.542 | 0.571946 | 0.4495 |
|  | serialseq2 | serialseq1 | + Condition | serialseq2 to serialseq1 | 7 | 351.8829 | 370.527 | -168.9415 | 7.201037 | 0.0073 |
|  | seriaseql3 | serialseq2 | + Group:Condition | serialseq3 to serialseq2 | 8 | 353.6076 | 374.9151 | -168.8038 | 0.275324 | 0.5998 |
| serialseq2 equation: ***MeanSEQ ~ Group + Condition, random ~1\|Participant/Group/Condition*** | | | | | | | |  |  |  |
| **Model Summary** | **df** | **Beta** | **SE** | **t-value** | **p-value** |  |  |  |  |  |
| Intercept | 52 | 3.176331 | 0.14166303 | 22.421734 | 0 |  |  |  |  |  |
| Group | 51 | 0.105894 | 0.14166303 | 0.747508 | 0.4582 |  |  |  |  |  |
| Condition | 52 | -0.267363 | 0.09766057 | -2.737676 | 0.0085 |  |  |  |  |  |

***Table S1:3 Depth of Encoding***

| **Model specification** | **Model name** | **Nested Model** | **Fixed Effects** | **Model Comparison** | **Model fit** | | | | | |
| --- | --- | --- | --- | --- | --- | --- | --- | --- | --- | --- |
|  |  |  |  |  | **df** | **AIC** | **BIC** | **LogLik** | **L. Ratio** | **p-value** |
|  | | | | | | | | | | |
| Mean Accuracy (%) | Baseline |  |  |  | 7 | 3381.735 | 3410.083 | -1683.868 |  | |
|  | depth1 | Baseline | + Group | depth1 to Baseline | 8 | 3383.703 | 3416.101 | -1683.851 | 0.03222 | 0.8576 |
|  | depth2 | depth1 | + Condition | depth2 to depth1 | 9 | 3316.024 | 3352.472 | -1649.012 | 69.6783 | <.0001 |
|  | depth3 | depth2 | + Delay | depth3 to depth2 | 10 | 3162.999 | 3203.497 | -1571.5 | 155.02521 | <.0001 |
|  | depth4 | depth3 | + Depth | depth4 to depth3 | 11 | 3159.768 | 3204.315 | -1568.884 | 5.23107 | 0.0222 |
|  | depth5 | depth4 | + Group:Condition | depth5 to depth4 | 12 | 3157.638 | 3206.234 | -1566.819 | 4.13057 | 0.0421 |
|  | depth6 | depth5 | + Group:Delay | depth6 to depth5 | 13 | 3159.223 | 3211.87 | -1566.612 | 0.41433 | 0.5198 |
|  | depth7 | depth6 | + Group:Depth | depth7 to depth6 | 14 | 3158.468 | 3215.164 | -1565.234 | 2.75568 | 0.0969 |
|  | depth8 | depth7 | + Condition:Delay | depth8 to depth7 | 15 | 3151.499 | 3212.245 | -1560.75 | 8.96828 | 0.0027 |
|  | depth9 | depth8 | + Condition:Depth | depth9 to depth8 | 16 | 3147.175 | 3211.971 | -1557.588 | 6.32388 | 0.0119 |
|  | depth10 | depth9 | + Delay:Depth | depth10 to depth9 | 17 | 3148.883 | 3217.728 | -1557.441 | 0.29285 | 0.5884 |
|  | depth11 | depth10 | + Group:Delay:Depth | depth11 to depth10 | 18 | 3150.72 | 3223.615 | -1557.36 | 0.16248 | 0.6869 |
|  | depth12 | depth11 | + Group:Condition:Delay | depth12 to depth11 | 19 | 3151.923 | 3228.868 | -1556.961 | 0.79721 | 0.3719 |
|  | depth13 | depth12 | + Group:Condition:Depth | depth13 to depth12 | 20 | 3152.178 | 3233.173 | -1556.089 | 1.7446 | 0.1866 |
|  | depth14 | depth13 | + Condition:Delay:Depth | depth14 to depth13 | 21 | 3154.169 | 3239.213 | -1556.084 | 0.00937 | 0.9229 |
|  | depth15 | depth14 | + Group:Condition:Delay:Depth | depth15 to depth14 | 22 | 3155.359 | 3244.453 | -1555.68 | 0.80973 | 0.3682 |

depth9 equation:  ***MeanACC ~ Group + Condition + Delay + Depth + Group:Condition + Group:Delay + Group:Depth + Condition:Delay + Condition:Depth, random = ~1|Participant/Group/Condition/Delay/Depth***

| **Model Summary** | **df** | **Beta** | **SE** | **t-value** | **p-value** |
| --- | --- | --- | --- | --- | --- |
| (Intercept) | 209 | 31.76425 | 1.603 | 19.815326 | 0 |
| Group | 51 | -0.28436 | 1.603 | -0.177393 | 0.8599 |
| Condition | 51 | -8.67996 | 0.6954 | -12.482079 | 0 |
| Delay | 103 | -5.91056 | 0.3594 | -16.444148 | 0 |
| Depth | 209 | 0.77886 | 0.3594 | 2.166903 | 0.0314 |
| Group:Condition | 51 | 1.42421 | 0.6954 | 2.048063 | 0.0457 |
| Group:Delay | 103 | 0.23527 | 0.3594 | 0.654568 | 0.5142 |
| Group:Depth | 209 | 0.60524 | 0.3594 | 1.683889 | 0.0937 |
| Condition:Delay | 103 | -1.07704 | 0.3578 | -3.009937 | 0.0033 |
| Condition:Depth | 209 | -0.89361 | 0.3578 | -2.497298 | 0.0133 |
