# Supplementary material for: The morning after the night before: Alcohol-induced blackouts impair next day recall in sober young adults
Source: PLoS One. 2021 May 3;16(5):e0250827. doi: 10.1371/journal.pone.0250827 (PMC8092761; doi:10.1371/journal.pone.0250827)
Supplement: S2 Appendix — (DOCX) [file pone.0250827.s002.docx]

**S2 Appendix. Within MBO Group Analysis Model Output Tables**

***Table S2:1 Free Recall***

| **Model specification** | **Model name** | **Nested Model** | **Fixed Effects** | **Model Comparison** | **Model fit** | | | | | |
| --- | --- | --- | --- | --- | --- | --- | --- | --- | --- | --- |
|  |  |  |  |  | **df** | **AIC** | **BIC** | **LogLik** | **L. Ratio** | **p-value** |
|  | | | | | | | | | | |
| Mean Accuracy (%) | Baseline |  |  |  | 4 | 648.7356 | 658.3134 | -320.3678 |  | |
|  | freel1 | Baseline | + Condition | free1 to Baseline | 6 | 618.9457 | 633.3124 | -303.4728 | 33.78991 | <.0001 |
| free1 model equation: ***MeanACC ~ Condition, random = ~1\|Participant/Condition*** | | | | |  |  |  |  |  |  |
| **Model Summary** | **df** | **Beta** | **SE** | **t-value** | **p-value** |  |  |  |  |  |
| Intercept | 50 | 52.00168 | 1.649031 | 31.534697 | 0 |  |  |  |  |  |
| Condition - Before/After | 50 | -8.17027 | 1.345111 | -6.074048 | 0 |  |  |  |  |  |
| Condition - Before/AfterMBO | 50 | 1.19685 | 1.448267 | 0.826405 | 0.4125 |  |  |  |  |  |

***Table S2:2 Serial Recall: Mean Accuracy***

| **Model specification** | **Model name** | **Nested Model** | **Fixed Effects** | **Model Comparison** | **Model fit** | | | | | |
| --- | --- | --- | --- | --- | --- | --- | --- | --- | --- | --- |
|  |  |  |  |  | **df** | **AIC** | **BIC** | **LogLik** | **L. Ratio** | **p-value** |
|  | | | | | | | | | | |
| Mean Accuracy (%) | Baseline |  |  |  | 4 | 633.8919 | 643.4697 | -312.946 |  | |
|  | serial1 | Baseline | + Condition | serial1 to Baseline | 6 | 611.9743 | 626.341 | -299.9872 | 25.9176 | <.0001 |
| serial2 equation: ***MeanACC ~ Condition, random = ~1\|Participant/Condition*** | | | | |  |  |  |  |  |  |
| **Model Summary** | **df** | **Beta** | **SE** | **t-value** | **p-value** |  |  |  |  |  |
| Intercept | 50 | 46.47487 | 1.834484 | 25.334027 | 0 |  |  |  |  |  |
| Condition - Before/After | 50 | -6.78183 | 1.186782 | -5.714473 | 0 |  |  |  |  |  |
| Condition - After/AfterMBO | 50 | -3.98512 | 1.282463 | -3.107396 | 0.0031 |  |  |  |  |  |

***Table S2: 3 Depth of Encoding***

| **Model specification** | **Model name** | **Nested Model** | | | **Fixed Effects** | | **Model Comparison** | | **Model fit** | | | | | | | |  |
| --- | --- | --- | --- | --- | --- | --- | --- | --- | --- | --- | --- | --- | --- | --- | --- | --- | --- |
|  |  |  |  |  |  |  |  |  | **df** | | **AIC** | | **BIC** | **LogLik** | **L. Ratio** | **p-value** |  |
|  | | | | | | | | | | | | | | | | |  |
| Mean Accuracy (%) | Baseline |  | | |  | |  | | 6 | | 2557.885 | | 2580.569 | -1272.942 |  | |  |
|  | depth1 | Baseline | | | + Condition | | depth1 to Baseline | | 8 | | 2518.357 | | 2548.602 | -1251.178 | 43.52814 | <.0001 |  |
|  | depth2 | depth1 | | | + Delay | | depth2 to depth1 | | 9 | | 2417.606 | | 2451.633 | -1199.803 | 102.75014 | <.0001 |  |
|  | depth3 | depth2 | | | + Depth | | depth3 to depth2 | | 10 | | 2408.377 | | 2446.184 | -1194.188 | 11.22965 | 0.0008 |  |
|  | depth4 | depth3 | | | + Condition:Delay | | depth4 to depth3 | | 12 | | 2405.505 | | 2450.874 | -1190.753 | 6.8715 | 0.0322 |  |
|  | depth5 | depth4 | | | + Condition:Depth | | depth5 to depth4 | | 14 | | 2402.185 | | 2455.115 | -1187.092 | 7.32071 | 0.0257 |  |
|  | depth6 | depth5 | | | + Delay:Depth | | depth6 to depth5 | | 15 | | 2403.769 | | 2460.48 | -1186.884 | 0.4158 | 0.519 |  |
|  | depth7 | depth6 | | | + Condition:Delay:Depth | | depth7 to depth6 | | 17 | | 2407.445 | | 2471.717 | -1186.722 | 0.32416 | 0.8504 |  |
| depth5 equation: ***MeanACC ~ Condition + Delay + Depth + Condition:Delay + Condition:Depth, random = ~1\|Participant/Condition/Delay/Depth*** | | | | | | | | | | | | | | | | | |
| **Model Summary** | | | **df** | **Beta** | | **SE** | | **t-value** | | **p-value** | |  |  |  |  |  |  |
| (Intercept) | | | 159 | 29.528623 | | 1.9802001 | | 14.911939 | | 0 | |  |  |  |  |  |  |
| Condition - After/Before | | | 50 | 9.207009 | | 1.1433623 | | 8.052574 | | 0 | |  |  |  |  |  |  |
| Condition - After/AfterMBO | | | 50 | -3.902524 | | 1.2371711 | | -3.154393 | | 0.0027 | |  |  |  |  |  |  |
| Delay - Delay/Immediate | | | 78 | -5.031512 | | 0.4179765 | | -12.037786 | | 0 | |  |  |  |  |  |  |
| Depth - Deep/Shallow | | | 159 | -1.438031 | | 0.4179765 | | -3.440459 | | 0.0007 | |  |  |  |  |  |  |
| Condition After/Before:Delay | | | 78 | 0.146455 | | 0.5791653 | | 0.252872 | | 0.801 | |  |  |  |  |  |  |
| Condition After/AfterMBO: Delay | | | 78 | 1.287551 | | 0.6142975 | | 2.095972 | | 00393 | |  |  |  |  |  |  |
| Condition After/Before:Depth | | | 159 | -1.263118 | | 0.5791653 | | -2.180929 | | 0.307 | |  |  |  |  |  |  |
| Condition After/AfterMBO:Depth | | | 159 | -0.107863 | | 0.6142975 | | -0.175587 | | 080 | |  |  |  |  |  |  |
